# Neighborhood context and children's health care utilization and health outcomes: a comprehensive descriptive analysis of national survey data

**DOI:** 10.1093/haschl/qxad038

**Published:** 2023-08-24

**Authors:** Izabela E Annis, Neal A deJong, Robert B Christian, Scott A Davis, Phillip M Hughes, Kathleen C Thomas

**Affiliations:** Division of Pharmaceutical Outcomes and Policy, Eshelman School of Pharmacy, University of North Carolina at Chapel Hill, Chapel Hill, NC 27599, United States; Department of Pediatrics, University of North Carolina at Chapel Hill School of Medicine, Chapel Hill, NC 27599, United States; Department of Psychiatry, University of North Carolina at Chapel Hill School of Medicine, Chapel Hill, NC 27599, United States; Division of Pharmaceutical Outcomes and Policy, Eshelman School of Pharmacy, University of North Carolina at Chapel Hill, Chapel Hill, NC 27599, United States; Division of Pharmaceutical Outcomes and Policy, Eshelman School of Pharmacy, University of North Carolina at Chapel Hill, Chapel Hill, NC 27599, United States; Cecil G. Sheps Center for Health Services Research, Chapel Hill, NC 27599, United States; Division of Pharmaceutical Outcomes and Policy, Eshelman School of Pharmacy, University of North Carolina at Chapel Hill, Chapel Hill, NC 27599, United States; Cecil G. Sheps Center for Health Services Research, Chapel Hill, NC 27599, United States

**Keywords:** child opportunity index, children's health, children's healthcare utilization

## Abstract

While child health and health care disparities arising from unequal distribution of resources are well documented, a nationally representative inventory of health and well-being for children across the spectrum of opportunity is lacking. Using the nationally representative sample of children from pooled 2013–2017 Medical Expenditure Panel Survey data linked to the census-tract-level Child Opportunity Index 2.0, a composite measure of neighborhood health, education, and socioeconomic conditions, we describe US children's socioeconomic characteristics, health care utilization and expenditures across the spectrum of child neighborhood opportunity levels. We found that neighborhood level of child opportunity was associated with almost all of children's health status, health care utilization, expenditures, access to care, and satisfaction with care outcomes. Children living in lower-opportunity neighborhoods had the highest rates of poor physical and mental health status and fewest ambulatory care visits but accounted for the highest share of emergency department visits. Their parents were also least likely to report having positive experiences with health care, good communication with providers, and easy access to care. Our findings underscore the myriad harms to children of gaps in health, education, and financial resources at the community level and provide targets for public investments to improve child-focused outcomes.

## Introduction

The notion that neighborhood environment influences one's economic and health outcomes has been studied extensively.^1^ Recent research has examined the relationships between neighborhood-level characteristics and individual-level health and quality-of-life outcomes, strengthening the evidence that individuals’ physical and social environment influences their economic and health outcomes.^2^ Unequal distribution of resources discernible through variation in neighborhoods’ social, economic, and environmental conditions has been cited as a root cause of health inequity.^3^ It has been said that a person's zip code might be equally or more important to their health than their genetic code.^4^ Recognizing that social determinants of health have a major influence on people's health and quality of life and contribute to wide disparities and inequities in health, the US Department of Health and Human Services set a range of goals to improve the physical and social conditions of the neighborhoods where people live and work.^5^

Evidence of existing neighborhood effects on health has been demonstrated in diverse populations, including children. Neighborhoods where children live affect their near and distal health and well-being and can determine whether they thrive or struggle in all aspects of their adult lives.^6–10^ Accepting this fundamental premise that intertwined and complex neighborhood conditions can have a long-term effect on children's outcomes, Acevedo-Garcia and colleagues introduced^11^ and later updated^12^ a composite measure of child opportunity—the Child Opportunity Index (COI). The index captures 29 neighborhood attributes from 3 domains (education, health and environment, social and economic) that have been shown to specifically influence children's development and outcomes.^13^ The COI is a relative, national-level measure available for almost all US neighborhoods (census tracts). Census tracts approximate neighborhoods as they were established by the US Census Bureau to be as homogenous as possible with respect to sociodemographic characteristics.^14^

While several previous studies used this composite measure of neighborhood opportunity to examine associations between children's COI levels and specific health outcomes (pediatric asthma hospitalization, acute care use, emergency department utilization),^15–17^ the literature lacks a nationally representative inventory of health and well-being for children across the spectrum of COI levels. A broader understanding of health, health care utilization, access, and quality of care across COI levels could highlight areas of health and health care with inequalities that might stem from poor neighborhood conditions. Our intention was to lay the groundwork for future studies examining the relationship between pediatric health outcomes, utilization, and levels of neighborhood opportunity. Therefore, for this study we linked children's individual-level Medical Expenditure Panel Survey (MEPS) data to census-tract-level COI 2.0 scores. Our objective was to portray detailed socioeconomic, health, and health care utilization profiles of children living in US neighborhoods with varying levels of child opportunity. We examined demographic, family, health status, health care utilization, expenditures, access, and care satisfaction measures across the range of COI levels.

## Data and methods

### Data source and sample

In this retrospective, cross-sectional analysis, we used pooled MEPS data from the years 2013–2017. This time period was chosen to align with the data that were used in the construction of the COI 2.0. The MEPS is a nationally representative, longitudinal survey of health care utilization and spending, sponsored by the Agency for Healthcare Research and Quality.^18^ The MEPS provides national estimates of health care service use and costs, as well as data on individuals’ mental and physical health conditions and demographic and socioeconomic characteristics. The MEPS oversamples minority and low-income households and provides weights that account for the complex sampling design. In addition to publicly available MEPS Full Year Consolidated Data files, Medical Conditions files, and Prescribed Medicines event files, our study used restricted MEPS files accessible through the Federal Statistical Research Data Centers. Beginning in 2013, all MEPS respondents were geo-coded, and their census tract information can be used to merge external neighborhood-level variables. This research used individual-level data from MEPS linked to census-tract-level COI 2.0 data. The study sample included all children under 18 years of age. Because MEPS data are publicly available and de-identified, this study was exempt from institutional review board approval.

### Child opportunity levels

Neighborhood child opportunity in terms of health, environment, quality of education, and social and economic resources was measured with the COI 2.0.^13^ The index is a weighted average of 29 indicators from 3 domains weighted to reflect the magnitude of the indicator’s predictive power of children's health and economic outcomes. The index and its components are available for most census tracts in the 50 US states and Washington, DC. Higher values of the index indicate higher levels of opportunity. In this study, we used nationally normed COI levels,^13^ an ordered, categorical variable with 5 levels ranging from “very low” to “very high” opportunity. The levels were determined by calculating the cutoff points based on the distribution of the overall index weighted by child population estimates from the 2017 American Community Survey (ACS). Consequently, each level includes 20% of the US child population.

### Measures

Acknowledging that health is a multidimensional construct, we selected a wide range of demographic and economic characteristics to describe the study sample and included an extensive list of health-related outcomes to describe population-level children's health patterns across levels of neighborhood opportunity. We included child- and family-level socioeconomic measures to explore the construct validity of the COI. Specifically, we used the MEPS Full Year Consolidated data files to obtain the following: age (0–5, 6–11, and 12–17 y), sex, race/ethnicity (Hispanic, non-Hispanic [NH] American Indian/Alaska Native, NH Asian/Hawaiian/Pacific Islander, NH Black, NH multiple races, and NH White), census region, insurance status (any private, public only, uninsured). Our measure of race is a social construct reflecting the experience of racism. Family measures include family size (1–2, 3–5, 6+), single-parent household, highest level of education, and income. Income was categorized in relation to the federal poverty level (FPL): poor/near poor (<125% of FPL), low-income (125% to <200% of FPL), middle-income (200% to <400% of FPL), and high-income (≥400% of FPL). The calculation of FPL was controlled for family size and age of the head of the family.

Because parents are responsible for health care seeking and advocating for their children and because of its known influence on health care utilization and outcomes,^19^ we included a measure of adult health literacy. In lieu of an individual-level health literacy measure, which is not available in our data, we included a neighborhood-level health literacy variable. Adult health literacy was captured at the census tract level and is based on a predictive model utilizing demographic and socioeconomic variables from the US Census Bureau and the ACS.^20,21^ The model produces average estimates of neighborhood-level health literacy that are in agreement with scores used by the 2003 National Assessment of Adult Literacy (NAAL).^22^ Individuals with an NAAL health literacy score of 225 or less are described as having “basic or low” health literacy, indicating that “they have difficulty obtaining, processing, and understanding basic health information and services.” Our study categorized neighborhood health literacy estimates according to the 2003 NAAL criteria of “above basic” (score >225) and “basic/below basic” (score ≤225).

Child medical need was measured by parent-reported physical and mental health status (poor/fair/good, very good/excellent). We included indicators for 3 common and concerning chronic childhood conditions: attention-deficit hyperactivity disorder/attention-deficit disorder (ADHD/ADD), asthma, and diagnosis of any mental health or behavioral problem.^23^

We included all standard MEPS measures of health care utilization and expenditures,^18^ including office-based, outpatient, emergency room (ER), hospital, dental, home health, and prescribed medicines utilization and expenses. The MEPS measure of home health provider days includes days provided by paid providers and informal caregivers. All expenditures were adjusted for inflation to 2017 dollars using the Personal Health Care Index.^24^

We captured access to health care and health care quality. We included whether a child has a usual source of care provider and whether that provider is a medical doctor. Using questions from the Consumer Assessment of Healthcare Providers and Systems (CAHPS), we constructed access to care and communication with physician composite scores. The access-to-care composite included 2 items (“How often a person got care as soon as needed” and “How often a person got an appointment as soon as needed”). The communication with physician composite included 4 items (“How often doctors explained things,” “How often doctors listened carefully,” “How often doctors showed respect,” “How often doctors spent enough time”). Responses were coded on a 4-point Likert scale ranging from 1 (never) to 4 (always). Composites were calculated as means across items. To discriminate better between COI levels, we dichotomized all measures to indicate a positive response.^25^ Scores of 4 (always) were considered positive. A global rating of quality of health care providers ranged from 0 (worst health care possible) to 10 (best health care possible). In our analyses, the global rating was also dichotomized (8–10 vs 0–7) to reflect a positive experience.

### Statistical analysis

We used descriptive statistics and generated national estimates of proportions and means and their corresponding standard errors (SEs) to characterize the profiles of children living across the spectrum of COI levels. We also estimated total annual expenditures from each of the main health care categories. To examine whether there was an association between the measured characteristics and neighborhood COI, we used Rao-Scott chi-square tests for categorical variables and analysis of variance for continuous measures.

Since our analyses utilized pooled MEPS data from the years 2013–2017 to generate estimates that reflect national average annual numbers, the person-weight variables were divided by 5 (number of years of data being pooled). To account for complex survey design and repeated measures arising from overlapping panel design, we used stratum and cluster variables and appropriate SURVEY procedures in SAS version 9.4 (SAS Institute, Cary, NC).

In supplementary analyses, we compared the distribution of children's race/ethnicity data across the range of COI levels in the MEPS database to the corresponding numbers in the 2017 ACS data that were used in the development of the COI (Figure S1).

## Results

The total sample included 45 092 children (50.9% male; mean [SE] age: 8.7 [0.1] y) representing, on average, 73 953 689 US children annually across the years 2013–2017. Table 1 presents demographic, socioeconomic, and health status characteristics of US children stratified by COI levels. Children living in very low compared with very high COI neighborhoods were 32.3% vs 4.0% NH Black, 20.7% vs 68.2% NH White, and 38.4% vs 10.9% Hispanic. Almost 44% vs 31.3% of children from very low compared with very high COI neighborhoods lived in the South and 68.6% vs 12% had only public insurance. Over 22% vs 12.8% of children in very low compared with very high COI neighborhoods lived in large families (family size ≥6), but they were also most likely to live in a single-parent households (39.0% vs 12.3%). COI levels were strongly associated with community-level health literacy. In very low compared with very high COI neighborhoods, 48.5% vs 100% of children lived in areas where average health literacy skills were above basic.

**Table 1.** Sociodemographic and health status characteristics of all US children, in total and stratified by Child Opportunity Index levels, 2013–2017.

Characteristics	Very low	Low	Moderate	High	Very high	Total	*P*
Unweighted n	13 660	10 741	7721	6877	6093	45 092	
Weighted n (%)	14 420 086 (19.5)	14 245 597 (19.3)	14 377 268 (19.4)	14 943 493 (20.2)	15 967 245 (21.6)	73 953 689	
Age group (y)							<.0001
0–5	34.0 (0.7)	34.0 (1)	30.9 (0.9)	32.5 (1.2)	30.9 (1.3)	32.4 (0.4)	
6–11	32.8 (0.7)	32.8 (0.7)	33.1 (0.9)	32 (0.9)	34.2 (1.0)	33.0 (0.4)	
12–17	33.3 (0.6)	33.2 (0.9)	36.0 (1.1)	35.5 (1.2)	34.9 (1.4)	34.6 (0.5)	
Gender							.9862
Female	48.7 (0.7)	49.2 (0.9)	49.4 (0.9)	49.0 (0.9)	49.3 (1)	49.1 (0.4)	
Male	51.3 (0.7)	50.8 (0.9)	50.6 (0.9)	51.0 (0.9)	50.7 (1)	50.9 (0.4)	
Race/ethnicity							<.0001
Hispanic	38.4 (2.6)	37.4 (2.4)	22.8 (1.5)	16.2 (1.2)	10.9 (0.9)	24.7 (1.1)	
Non-Hispanic American Indian/Alaska Native	0.7 (0.4)	0.9 (0.4)	0.6 (0.3)	0.4 (0.2)	—	0.5 (0.2)	
Non-Hispanic Asian/Pacific Islander	2.2 (0.5)	2.5 (0.3)	3.5 (0.6)	6.3 (0.8)	10.5 (0.9)	5.1 (0.3)	
Non-Hispanic Black	32.3 (2.3)	16.4 (1.2)	10.1 (0.9)	7.0 (0.7)	4.0 (0.6)	13.7 (0.8)	
Non-Hispanic multiple races	5.6 (0.7)	4.8 (0.5)	6.1 (0.6)	5.9 (0.7)	6.5 (0.8)	5.8 (0.4)	
Non-Hispanic White	20.7 (1.8)	38.1 (2.4)	56.8 (2)	64.3 (1.8)	68.2 (1.7)	50.1 (1.3)	
Census region							<.0001
Northeast	17.5 (1.6)	9.0 (1.3)	13.7 (1.5)	16.6 (1.9)	23.3 (2.3)	16.2 (0.7)	
Midwest	16.5 (1.7)	18.9 (1.7)	19.4 (2)	27.7 (2.2)	22.9 (2.3)	21.2 (0.9)	
South	43.5 (2.7)	44.0 (2.2)	43.9 (3.3)	30.4 (2.2)	31.3 (2.6)	38.4 (1.2)	
West	22.5 (3.1)	28.1 (2.0)	23.0 (2.3)	25.3 (2.2)	22.5 (2.7)	24.3 (1.0)	
Insurance							<.0001
Any private	26.9 (1.4)	41.4 (1.6)	60.2 (2.0)	71.0 (1.3)	86.0 (1.0)	57.8 (1.0)	
Public only	68.6 (1.4)	54.3 (1.6)	35.2 (2.0)	25.9 (1.3)	12.0 (1.0)	38.5 (1.0)	
Uninsured all year	4.4 (0.4)	4.3 (0.4)	4.6 (0.5)	3.1 (0.5)	2.0 (0.3)	3.7 (0.2)	
Family size							<.0001
1–2	7.4 (0.5)	7.8 (0.6)	5.4 (0.5)	5.5 (0.5)	2.9 (0.3)	5.7 (0.2)	
3–5	70.1 (1.2)	72.9 (1.1)	78.5 (1.3)	81 (1.4)	84.4 (1.6)	77.5 (0.7)	
6+	22.4 (1.3)	19.4 (1.2)	16.1 (1.2)	13.5 (1.4)	12.8 (1.6)	16.7 (0.7)	
Single-parent household	39.0 (1.6)	29.3 (1.1)	21.4 (1.2)	20.2 (1.2)	12.3 (1.1)	24.2 (0.7)	<.0001
Family highest educational level: college degree	14.4 (0.9)	23 (1.3)	38.7 (1.6)	52.5 (1.5)	76.4 (1.5)	41.9 (0.9)	<.0001
Family income as % of poverty line							<.0001
Poor	42.6 (1.2)	28.3 (1.3)	14.8 (1)	10.2 (0.7)	5.0 (0.5)	19.8 (0.7)	
Near poor	9.1 (0.5)	8.2 (0.6)	5.8 (0.5)	3.4 (0.3)	1.9 (0.3)	5.6 (0.2)	
Low-income	20.8 (0.7)	20.4 (1.0)	17.6 (1.0)	14.4 (0.9)	7.6 (0.7)	15.9 (0.4)	
Middle-income	21.3 (1.1)	28.5 (1.1)	36.8 (1.2)	34.1 (1.2)	26.0 (1.4)	29.3 (0.6)	
High-income	6.2 (0.6)	14.5 (1)	25.0 (1.6)	37.9 (1.3)	59.6 (1.8)	29.4 (0.7)	
Community health literacy score above basic	48.5 (2.3)	80.3 (2.6)	96.3 (0.8)	99.8 (0.1)	99.9 (0.1)	85.4 (1)	<.0001
Medical conditions							
ADHD/ADD diagnosis (age 5–17)	13.2 (0.7)	11.2 (0.9)	11.5 (0.8)	12.4 (0.7)	9.5 (0.7)	11.7 (0.8)	.0166
Asthma diagnosis	13.6 (0.6)	11 (0.6)	10.3 (0.7)	9.8 (0.7)	9.0 (0.7)	10.7 (0.4)	<.0001
Psychiatric condition	8.9 (0.5)	8.7 (0.6)	11.1 (0.7)	12.0 (0.7)	10.4 (0.6)	10.2 (0.3)	.0004
Physical health status							<.0001
Excellent/very good	59.7 (0.9)	66.7 (1.0)	71.5 (1.3)	74.4 (1.1)	80.2 (1.3)	70.7 (0.6)	
Poor/fair/good	40.3 (0.5)	33.3 (0.5)	28.5 (0.7)	25.6 (0.5)	19.8 (0.5)	29.3 (0.3)	
Mental health status							<.0001
Excellent/very good	61.2 (1.0)	68.7 (1.1)	71.4 (1.2)	74.1 (1.2)	80.2 (1.4)	71.3 (0.6)	
Poor/fair/good	38.8 (0.5)	31.3 (0.5)	28.6 (0.6)	25.9 (0.5)	19.8 (0.5)	28.7 (0.3)	

Abbreviation: ADHD/ADD, attention-deficit hyperactivity disorder/attention-deficit disorder.

Values are weighted % (SE). Source: Authors’ analysis of 2013–2017 data from Medical Expenditures Panel Survey and Child Opportunity Index 2.0 data. *P* values based on Rao-Scott chi-Square tests. “—” Indicates data masked due to small cell sizes.

Medical conditions and parent-reported health status also were associated with COI. Children from very low compared with very high COI neighborhoods were more likely to have ADHD (13.2% vs 9.5%) and asthma (13.6% vs 9.0%), and less likely to be diagnosed with a psychiatric condition (8.9% vs 10.4%). Their parents were less likely to report that their child was in excellent or very good physical (59.7% vs 80.2%) and mental (61.2% vs 80.2%) health.

**Figure 1.**
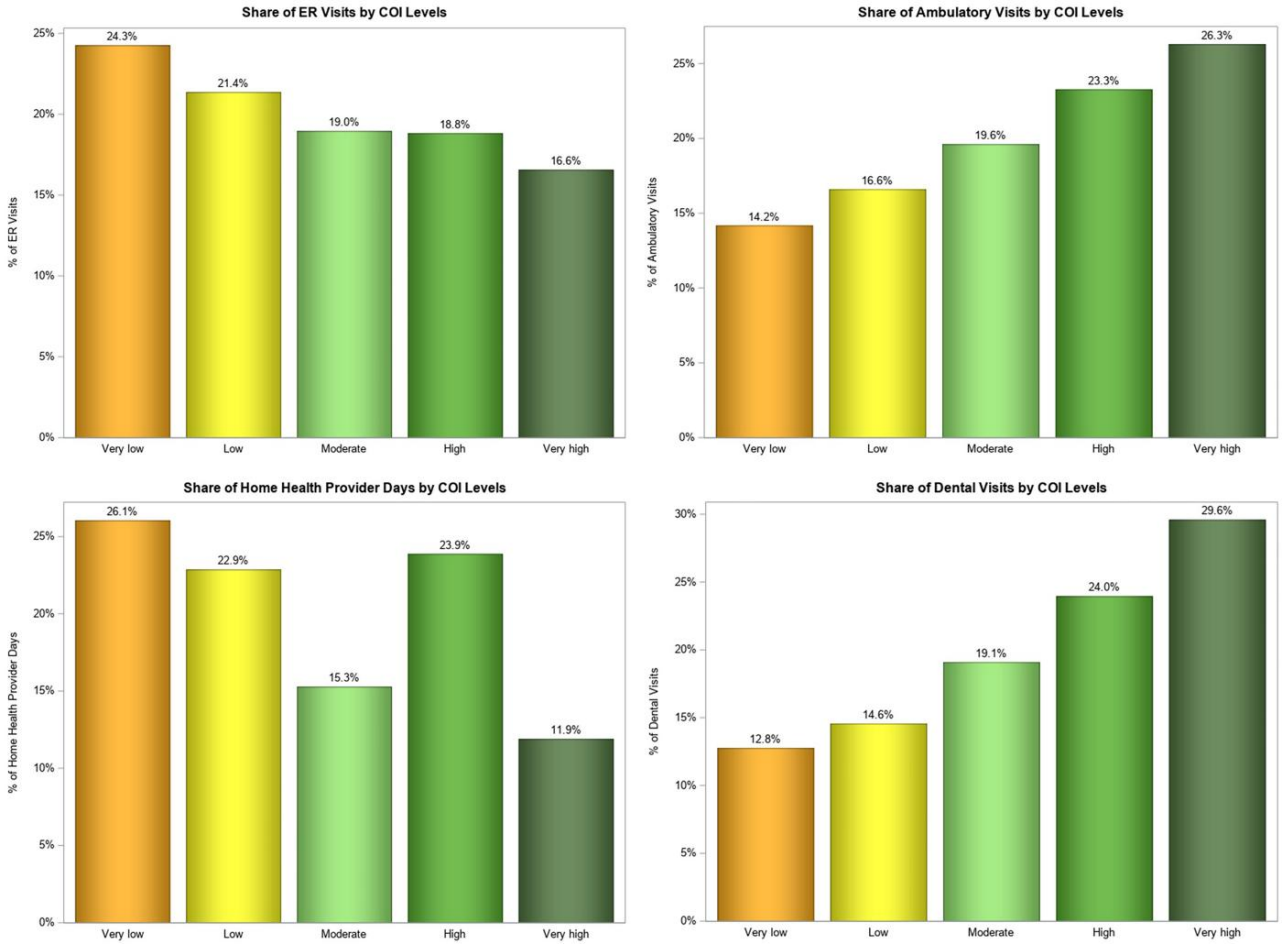
Shares of children's ER visits, ambulatory visits, home health provider days, and dental visits by COI levels, 2013–2017. Source: Authors’ analysis of 2013–2017 data from Medical Expenditures Panel Survey and COI 2.0 data. Abbreviations: COI, Child Opportunity Index; ER, emergency room.

**Figure 2.**
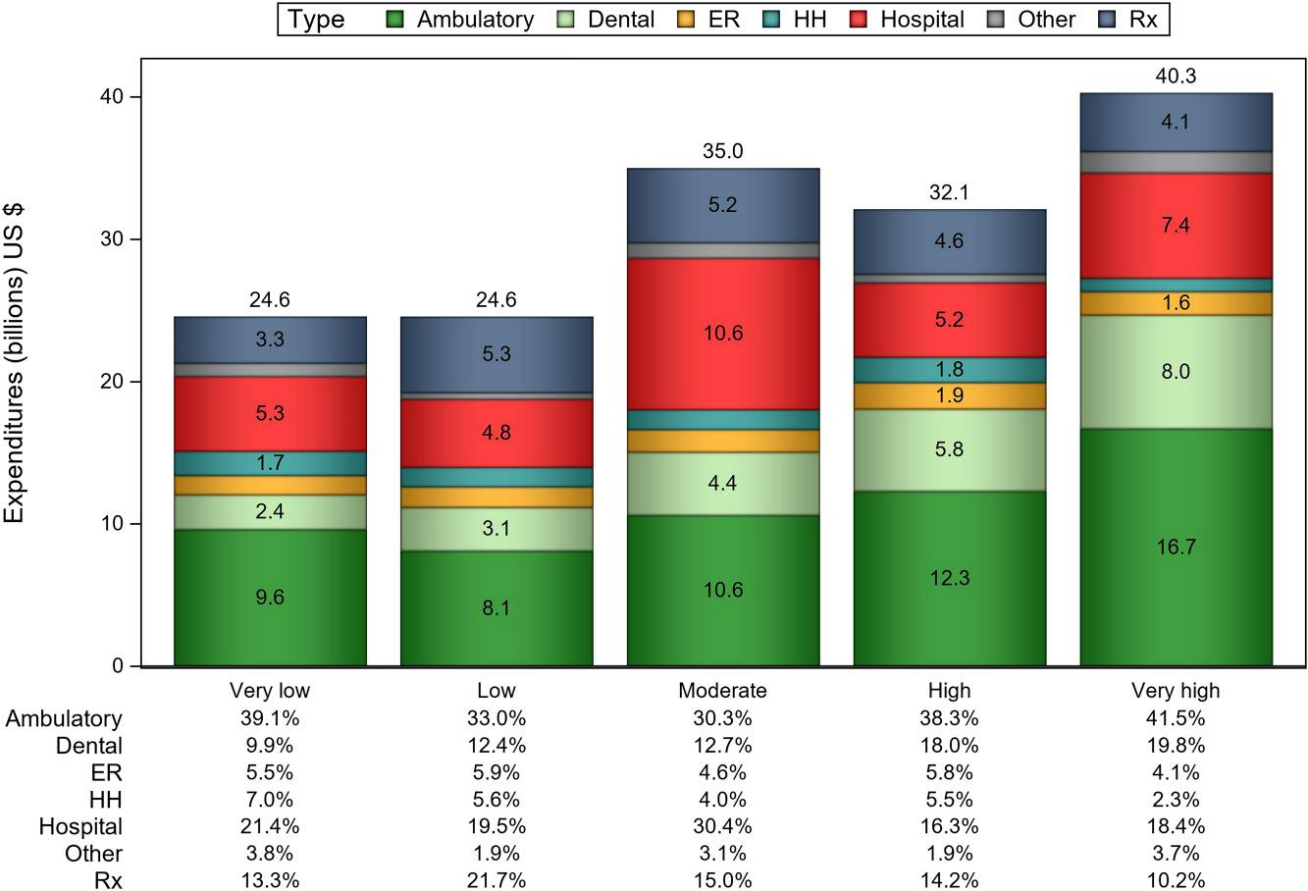
Average annual total children's health care expenditures and sources of expenditures, by Child Opportunity Index levels, 2013–2017. Source: Authors’ analysis of 2013-2017 data from Medical Expenditures Panel Survey and Child Opportunity Index 2.0 data. Abbreviations: ER = emergency room; HH = home health; Rx = prescription drugs.

The estimates of health care utilization and expenditures are presented in Table 2. All unadjusted analyses revealed strong associations between health care utilization, expenditures, and COI. An increasing trend in the number of ambulatory visits and dental visits was observed for increasing levels of COI. In contrast, a decreasing number of ER visits and home health provider days was associated with increasing levels of COI. Children living in very low compared with very high COI neighborhoods, on average, had the fewest ambulatory visits (mean [SE]: 2.7 [0.2] vs 4.5 [0.2]) and dental visits (0.7 [0.0] vs 1.6 [0.1]), but the most ER visits (0.3 [0.0] vs 0.1 [0.0]) and home health provider days (0.7 [0.2] vs 0.3 [0.1]) per year. In terms of share of health care utilization, even though similar percentages of all US children live in very low and very high COI areas (19.5% and 21.6%, respectively), children from very low compared with very high COI neighborhoods accounted for the lowest proportion of ambulatory visits (14.2% vs 26.3%), the fewest dental visits (12.8% vs 29.6%), and the most ER visits (24.3% vs 16.6%) and home health provider days (26.1% vs 11.9%) (Figure 1). Children from very low COI compared with very high COI neighborhoods had the lowest average ($1704 [$288] vs $2523 [$194]) and total ($24.6 billion vs $40.3 billion) annual health care expenditures (Figure 2).

**Table 2.** Health care utilization, expenditures, access, and satisfaction for all US children, in total and stratified by Child Opportunity Index levels, 2013–2017.

Characteristics	Very low	Low	Moderate	High	Very high	Total	*P*
Healthcare utilization, mean (SE), n/year							
Ambulatory visits	2.7 (0.2)	3.2 (0.2)	3.7 (0.2)	4.3 (0.2)	4.5 (0.2)	3.7 (0.1)	<.0001
Office-based visits	2.5 (0.1)	3.0 (0.2)	3.5 (0.2)	4.1 (0.2)	4.3 (0.2)	3.5 (0.1)	<.0001
Outpatient visits	0.2 (0.0)	0.2 (0.0)	0.2 (0.0)	0.2 (0.0)	0.2 (0.0)	0.2 (0.0)	<.0001
Emergency room visits	0.3 (0.0)	0.2 (0.0)	0.2 (0.0)	0.1 (0.0)	0.1 (0.0)	0.2 (0.0)	<.0001
Hospital discharges	0.03 (0.0)	0.04 (0.0)	0.03 (0.0)	0.03 (0.0)	0.03 (0.0)	0.03 (0.0)	<.0001
Hospital nights	0.20 (0.0)	0.24 (0.1)	0.30 (0.1)	0.17 (0.0)	0.13 (0.0)	0.21 (0.0)	<.0001
Prescribed medicines	2.2 (0.1)	2.4 (0.1)	2.3 (0.1)	2.5 (0.1)	2.0 (0.1)	2.3 (0.1)	<.0001
Dental visits	0.7 (0.0)	0.9 (0.0)	1.1 (0.0)	1.3 (0.1)	1.6 (0.1)	1.1 (0.0)	<.0001
Home health provider days	0.7 (0.2)	0.6 (0.1)	0.4 (0.01)	0.6 (0.1)	0.3 (0.1)	0.5 (0.1)	<.0001
Health care expenditures, mean (SE), $/year							
Total	1704.4 (287.9)	1724.8 (151.5)	2433.9 (355.0)	2148.8 (109.6)	2523.2 (193.9)	2116.6 (106.3)	<.0001
Office-based	379.4 (22.6)	468.1 (37.8)	573.7 (30.9)	684.2 (37.9)	855.8 (53.6)	598.7 (18.6)	<.0001
Outpatient	286.7 (215.8)	100.5 (13.4)	163.8 (25.9)	138.3 (21.9)	190.3 (27.9)	176.1 (42.8)	<.0001
Emergency room	93.9 (7.6)	101.5 (8.1)	111.8 (9.7)	124.8 (11.5)	102.9 (8.4)	107.0 (3.9)	<.0001
Hospital	365.5 (72.8)	337.1 (54.1)	740.2 (271.8)	349.8 (64.0)	463.2 (146.1)	450.8 (65.1)	<.0001
Prescribed medicines	227.1 (17.3)	374.7 (123.8)	364.3 (95.8)	305.4 (29.6)	258.4 (30.8)	304.8 (31.4)	<.0001
Dental care	168.8 (12.2)	214.2 (13.5)	308.0 (19.8)	387.3 (22.5)	499.8 (32.7)	320.2 (10.6)	<.0001
Home health care	118.7 (34.6)	96.4 (24.3)	97.5 (30.3)	118.8 (39.3)	58.9 (15.6)	97.4 (13.2)	<.0001
Other medical	64.4 (41.3)	32.2 (5.1)	74.6 (40.6)	40.0 (4.5)	93.9 (45.3)	61.6 (15.3)	<.0001
Access, communication, and quality of health care, % (SE)							
Has usual source of care provider	89.8 (0.8)	91.1 (0.6)	92.8 (0.6)	93.8 (0.5)	95.2 (0.4)	92.6 (0.3)	<.0001
Usual source of care provider is a medical doctor	29.3 (1.3)	32.7 (1.5)	36.2 (1.9)	36.2 (1.6)	37.1 (2.0)	34.5 (1.0)	.0012
Easy access to care	79.1 (1.0)	81.1 (1.0)	83.0 (1.0)	84.5 (1.0)	84.0 (1.0)	82.6 (0.5)	<.0001
Good communication with health care provider	81.8 (0.8)	81.3 (0.9)	83.0 (1.1)	85.3 (0.8)	85.8 (0.9)	83.6 (0.4)	<.0001
Highest global rating of health care providers	88.9 (0.6)	90.0 (0.6)	91.6 (0.6)	91.4 (0.6)	93.9 (0.6)	91.3 (0.3)	<.0001

Source: Authors’ analysis of 2013–2017 data from Medical Expenditures Panel Survey and Child Opportunity Index 2.0 data.

Average reports of access, communication, and satisfaction with health care were high overall, but parents reported progressively higher levels of access, communication, and satisfaction with health care as level of COI increased. Seventy-nine percent vs 84.0% of parents of children from very low compared with very high COI neighborhoods reported easy access to care, 81.8% vs 85.8% reported good communication with the provider, and 88.9% vs 93.9% had the highest global rating (Table 2).

Our supplementary analyses (Figure S1) comparing the distribution of children by race/ethnicity across the spectrum of COI levels in the MEPS data with the 2017 ACS data showed that overall patterns were similar with the distributions within the very low COI category being almost identical and the distributions within the low COI being most disparate.

## Discussion

In this national cross-sectional study, we examined the association between children's neighborhood opportunity levels and an array of pediatric health-related outcomes. We found evidence of racial, health status, health care utilization, access, and satisfaction with care inequalities. Children living in low COI areas were more often minority and living in large families where adults most often had basic or below basic health literacy skills. In contrast, their counterparts living in the higher opportunity areas were predominantly White and living in families with private insurance and above basic health literacy skills. Even though children in very low COI neighborhoods were least likely to be in excellent/very good mental and physical health, their average annual and total health care expenditures were the lowest of all COI levels.

Our findings echo the original work on COI 2.0 by Acevedo-Garcia et al^12^ that highlighted striking racial inequities in neighborhood conditions. Their analyses of metropolitan areas also found that the South had the lowest COI scores, which is corroborated by our analyses. Our study found substantial evidence in support of the construct validity of the COI. Our results show that neighborhood levels of child opportunity are associated with various individual-level socioeconomic factors, such as coverage by public insurance, income, parental education, family size, and community health literacy scores.

Our study draws attention to inequities in mental and physical health status associated with and potentially arising from the conditions of the neighborhoods where children live. Our findings align with a study by Leventhal and Brooks-Gunn^26^ that demonstrated significant impacts of neighborhood poverty on parents’ and children's health outcomes by means of a randomized controlled trial. Our results also support findings from earlier research by Roubinov et al,^27^ in which low family socioeconomic status was associated with poor health among children living in low COI neighborhoods, and by Moore et al,^28^ which suggested that children living in neighborhoods with few amenities and more problems were less likely to be healthy. Consistent with prior work,^15^ we found that the prevalence of asthma increased with the gradient of COI. While we also found significant associations between levels of COI and rates of ADHD and psychiatric conditions, these relationships were not linear.

A striking finding from this study was the association between COI and children's use of several health care services. Other studies have demonstrated that children living in lower COI neighborhoods were more likely to utilize ERs than children living in higher COI areas.^16,17^ In an ecological study of pediatric asthma hospitalizations, Beck et al^15^ found that COI was associated with population-level asthma utilization rates. Our findings complement and build on this research, highlighting that children from lower COI neighborhoods account for a disproportionately high amount of ER visits and home health provider days, but utilize ambulatory and dental care much less frequently than their peers in higher opportunity neighborhoods. Our study illustrates that children living in lower COI neighborhoods have higher home health care utilization rates than children in higher COI neighborhoods. This finding may reflect that children living in lower COI areas experience a disproportionate burden of disability and, considering that they primarily rely on public sources of health insurance, this result is consistent with earlier reports showing that the majority of pediatric home health care is paid by Medicaid.^29^ Additional research should explore how home health care use varies by type of provider (paid vs informal caregivers) and neighborhood context.

Our study also shows that health care expenditures vary by COI. Children in very high COI neighborhoods had average annual per capita total health care expenditures almost 50% higher ($2523) than children in very low COI areas ($1704). Finally, total health care expenditures for all children living in very high COI neighborhoods exceeded $40 billion, with the largest share and the largest absolute expenditures spent on ambulatory and dental care, while children living in very low COI neighborhoods had the lowest total expenditures (∼$24.6 billion). These findings suggest that, despite having greater social and medical need, children in lower COI neighborhoods may have limited access to preventive services and primary care that are meant to keep them well and away from potentially preventable ER visits and hospitalizations. Meanwhile, the largest share of children's health care dollars is spent in socioeconomically advantaged neighborhoods, where children have access to many other, nonmedical supports and services and their families can more likely afford higher quality health care with better access to new, more effective, and potentially more costly treatments and services.

This imbalance between need and expenditures demonstrated by our findings may help explain why the United States continues to have the highest health care spending but the worst health outcomes of any high-income country.^30,31^ There is a critical need for continuous assessment of how health care resources are allocated to better promote equitable access and quality of care in order to reduce unmet need.

Last, our study found significant differences in measures of access, communication, and quality of care across the COI levels. Even though earlier studies investigated the relationship between access to care and COI,^32^ to our knowledge, this is the first study to shed light on whether the level and quality of children's resources in the neighborhood are associated with their parents’ communication and satisfaction with providers. While average rates were fairly high even in low opportunity areas, findings indicate that children in low opportunity areas experience critical disparities in use of care settings that potentially provide preventive and primary care and in overall expenditures. Combined, these results raise the concern as to whether families living in lower opportunity neighborhoods, often with high concentrations of poverty and limited resources, may compare themselves to families in their own neighborhoods and possibly be unaware that parents and children in higher opportunity neighborhoods have better access and quality of care. Previous research indicates that individuals are influenced by the social environment in which they live and can adjust their expectations based on cultural values, experiences, and context of care.^33^ While the investigation of the delays and reasons for not being able to get timely health care is beyond the scope of this work, future research should work to interpret these paradoxical findings. Future work must explore the value of the quality-of-care metrics for different types of families as well as new measures, such as therapeutic alliance,^34^ trust,^35^ and belief in the value of health care services,^36^ to deepen our understanding of current barriers to good health for US children.

The major strength of this study is its use of nationally representative children's data linked to neighborhood-level child opportunity. There are also important limitations to consider. First, the majority of information is self-reported and may be subject to recall and underreporting bias. Second, this study uses observational data and is descriptive in nature; as such, it is subject to confounding by observed and unobserved covariates and reverse causation. Further rigorous research is needed to assess whether or not neighborhood COI impacts children's health care use and health outcomes and if the financial and time burdens of children's health and health care use drive families to lower COI neighborhoods. Finally, while this study provides a comprehensive summary of COI neighborhood gradients and establishes a foundation for future research focusing on the COI, due to the scope of the study it lacks a detailed race/ethnicity equity analysis. Future research should examine neighborhood COI, race/ethnicity, and whether the neighborhood effects vary by race/ethnicity through the use of appropriate multilevel models.

## Conclusion

This 5-year, nationally representative study of US children found that children living in low COI neighborhoods were predominantly non-White, had the highest rates of poor physical and mental health status, and used fewer health care services, except for ER and home health services, compared with children from higher COI neighborhoods. The parents of children living in low COI neighborhoods were also least likely to report having positive experiences with health care, good communication with providers, and easy access to care. This research shows that, despite having substantial health care needs, children living in low COI neighborhoods have inadequate access to essential preventive, primary care services. These findings underscore the myriad harms to children of gaps in health, education, and financial resources at the community level and provide targets for public investments to improve child-focused outcomes.

## Supplementary Material

qxad038_Supplementary_Data
