# Supplementary material for: Neighborhood context and children's health care utilization and health outcomes: a comprehensive descriptive analysis of national survey data
Source: Health Aff Sch. 2023 Aug 24;1(3):qxad038. doi: 10.1093/haschl/qxad038 (PMC10986298; doi:10.1093/haschl/qxad038)
Supplement: qxad038_Supplementary_Data [file qxad038_Supplementary_Data.zip › COI and US Children Appendix.docx]

Appendix Figure 1. Comparison of the distribution of children by race/ethnicity across the levels of Child Opportunity Index in the Medical Expenditures Panel Survey data 2013-2017 to 2017 5-year American Community Survey data.


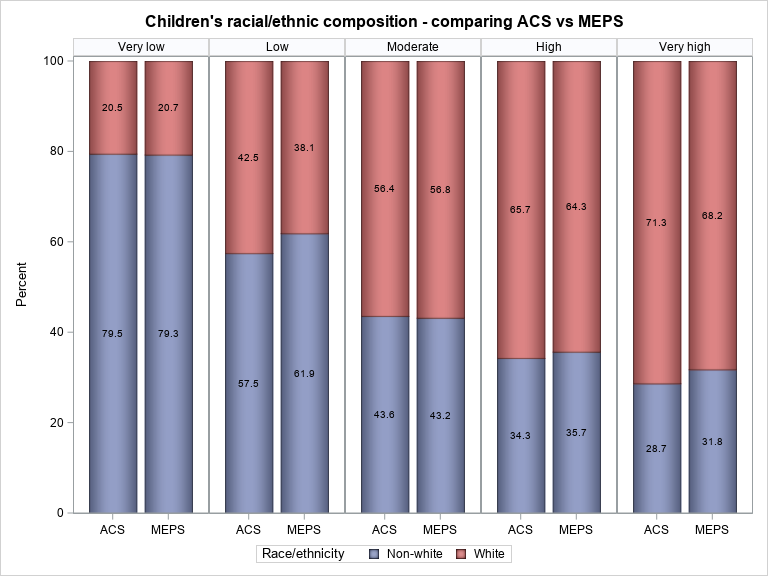


Source: Authors analysis of 2013 – 2017 data from Medical Expenditures Panel Survey and Child Opportunity Index 2.0 data. Notes: Non-white race/ethnicity group includes Hispanic, non-Hispanic (NH) Black, NH Asian/Native Hawaiian/Pacific Islander, NH American Indian/Alaska Native and NH multiple race children.
